# Improving Coral Grow-Out Through an Integrated Aquaculture Approach

**DOI:** 10.1155/anu/1446195

**Published:** 2025-04-13

**Authors:** Rachel C. Neil, Jonathan A. Barton, Andrew Heyward, David S. Francis, Leo Nankervis, Thomas S. Mock, David G. Bourne, Craig Humphrey

**Affiliations:** ^1^College of Science and Engineering, James Cook University, Townsville, Queensland, Australia; ^2^Australian Institute of Marine Science, Cape Cleveland, Queensland, Australia; ^3^AIMS@JCU, James Cook University, Townsville, Queensland, Australia; ^4^The National Sea Simulator, Australian Institute of Marine Science, Cape Cleveland, Queensland, Australia; ^5^Australian Institute of Marine Science, Indian Ocean Marine Research Centre, University of Western Australia, Crawley, Western Australia, Australia; ^6^Nutrition and Seafood Laboratory (NuSea.Lab), School of Life and Environmental Sciences, Deakin University, Queenscliff, Victoria, Australia; ^7^Centre for Sustainable Tropical Fisheries and Aquaculture, James Cook University, Townsville, Queensland, Australia

**Keywords:** aquaculture, coral, fish, nutrient enrichment, symbionts

## Abstract

Some coral species in natural reef systems derive benefits from fish which live in close association with them. This study investigates the benefits of incorporating fish in ex situ coral culture to enhance coral physiological performance. Corals that typically have fish associations (*Acropora kenti* and *Pocillopora verrucosa*) and those that do not (*Porites lutea* and *Platygyra daedalea*) were grown in aquaria under different fish-associated treatments for 3 months. Physiological performance of the corals, including growth, protein content, symbiont density and photosynthetic efficiency were assessed in the different treatments where corals were (1) kept with a school of *Chromis viridis* fed a pelleted diet, (2) supplied filtered water from a tank housing *C. viridis*, (3) fed live feeds whilst maintained with *C. viridis*, (4) supplied only with live feeds, (5) supplied with a pelleted fish diet without *C. viridis* and (6) not supplied feeds and without *C. viridis*. Whilst the responses of the corals varied between species, generally, exposure to fish or fish-water increased the protein and/or symbiont density within coral tissue. *A. kenti* and *P. lutea*, which derive a higher proportion of their energy requirement from autotrophy, displayed improved growth in the fish treatments, whilst the more heterotrophic *P. verrucosa* grew fastest when supplied with live feeds. The more heterotrophic, slow-growing *P. daedalea* did not show significant improvements in growth under any of the treatments, and there were no major differences in photosynthetic efficiency between treatments in any of the corals. These results indicate that incorporating fish into coral culture could provide an accessible source of nitrogen and phosphorous enrichment via the dissolved portion of the fish's wastes and, in turn, enhance the growth of corals more reliant on autotrophy, like Acroporids. The results point to potential efficiency gains for coral husbandry practices, with the aim of satisfying the growing demands of reef restoration and ornamental aquaculture.

## 1. Introduction

Integrated multi-trophic aquaculture is the practice of raising two or more types of organisms in proximity to each other, which may utilise the waste products of one species to act as nutritional input to the other [1]. Typically, a primary heterotrophic species such as a finfish is grown on a formulated diet, and then wastes and/or wastewater from this primary species are transferred to an extractive secondary species such as seaweed or a filter-feeding organism [2, 3]. Taking advantage of such relationships can allow farms to maximise production of both the primary and secondary species [4] whilst acquiring other benefits such as wastewater remediation [5–7] or an added price premium to integrated aquaculture products due to sustainability credentials or certifications [1, 8]. Other services, such as parasite control [9–11] or mediation of bacterial communities [12], may also be provided by the organisms cultured alongside the primary product.

Some coral species derive physiological effects from fish associations. For example, schooling juvenile *Haemulon* that reside over *Porites furcata* corals during the day resulted in increased coral growth rates compared to colonies of the same species with no fish present [13, 14]. Branching corals such as Pocilloporids often host damselfish (Pomacentridae) of various species [15] and may have faster growth rates [16–18], increased photosynthetic activity [19] and an increased resistance to bleaching [20, 21] compared to corals without fish present. Associated fish may also alleviate stressors caused by sedimentation on their hosts [22]. Such benefits may come from increased water movement resulting in higher oxygen and CO_2_ transport within the colony structure due to the physical behaviour of the fish, including fanning [19–21, 23, 24] or from nutrient enrichment in the form of both dissolved and particulate wastes from the fishes metabolic and digestive processes [13, 17, 18, 21, 25, 26].

Most Scleractinian coral species are thought to be mixotrophic but vary in their capacity and requirement for heterotrophic nutrient sources. One method to determine the relative rates of heterotrophic versus autotrophic energy acquisition in coral is to examine the overlap of δ^13^ C and δ^15^ N stable isotopes ratios in the coral host and their algal symbionts, with higher overlap indicating a higher reliance on autotrophic (i.e. symbiont derived) nutrition [27]. Using this method and supplementing with evidence such as fatty acid biomarkers, it has been determined that genera such as *Platygyra* and *Pocillopora* are more heterotopic, *Porites* mixotrophic and *Acropora* more autotrophic [28–30].

Production of corals in captive systems must increase if it is to meet the growing demand of the ornamental trade in addition to the emerging requirement of healthy individuals for out-planting to rehabilitate reefs. Consumer-derived nutrients (i.e. uptake of wastes from higher-trophic-order species by lower-order species) offer a potential mechanism to improve coral growth for restoration purposes, as demonstrated for in situ nurseries or at out-plant sites [31, 32]. The nutrition of corals kept in captivity for research or propagation remains an obstacle to long-term holding, as current commercial feeds are considered suboptimal [33]. Diets and feeding regimes should ideally be species specific, yet a lack of research has hindered their widespread suitability [34–36]. Integrating fish into captive coral culture may therefore contribute to a more holistic nutrition for corals, either from uptake of dissolved wastes for to support symbiont populations [37, 38] or heterotrophic feeding on egested particulate wastes [39, 40]. If reef restoration and ornamental coral aquaculture facilities aim to keep large coral colonies long-term as broodstock, the fanning of hosting damselfish may also be beneficial at preventing stagnant zones from forming in the deep inner branches where flow is reduced [41]. As such, this study aims to further elucidate such mechanisms of beneficial fish–coral interaction by evaluating coral performance either in association with schooling damselfish, the dissolved or particulate waste isolated from damselfish or conventional coral aquaculture diets, that is, mass-produced live feeds [42, 43]. Importantly, a range of coral species has been selected to span the autotrophic–heterotrophic energy acquisition spectrum and those with, and without, close fish associations.

## 2. Methodology

### 2.1. Experimental Setup

Five individual coral colonies collected from the reef >10 m apart (thereby likely representing different genotypes) of *Porites lutea*, *Platygyra daedalea*, *Pocillopora verrucosa* and *Acropora kenti* (formerly *Acropora tenuis* [44]) were collected from 2 to 7 m depth on Davies Reef (−18.825622, 147.626881) on the Great Barrier Reef, QLD, Australia. Corals were then brought to the Australian Institute of Marine Science's National Sea Simulator (SeaSim) in Cape Cleveland, QLD, Australia, where they were fragmented into ~10 g nubbins and allowed to heal for 1 month prior to the beginning of the experiment. During this period, corals were acclimated to experimental conditions including water flow, water temperature and light intensity (details below). Though different coral species have slightly different ideal environmental conditions, the parameters were all within acceptable culture conditions for these species, and represented a reasonable approximation of the conditions, corals may be kept in commercial aquaculture facilities. Ten randomly selected fragments from each colony were also collected and frozen at −20˚C when the corals first arrived at the facility (deemed ‘Field' samples) and after the 1-month acclimation period (deemed ‘Post-acclimation' samples) for subsequent analysis.

Twenty-eight 50 L experimental tanks, contained in water jackets to help control temperature, were set up and were randomly assigned one of the six experimental treatments, with four replicate tanks per treatment. The six treatments were: ‘Fish' corals kept in coculture with a school of 10 *Chromis viridis*, ‘Dissolved' corals supplied water filtered to 50 μm from a tank hosting a school of 10 *C. viridis*, ‘LiveFeeds + Fish' corals kept with 10 *C. viridis* whilst also given a supply of live feeds, ‘LiveFeeds' corals supplied only live feeds, ‘Pellets' corals supplied only the pelleted diet fed to the fish and ‘Control' corals kept with no fish and supplied no feeds. All *C. viridis* were juveniles (<5 cm total length) and were sourced from a commercial supplier and then 10 fish randomly assigned to each of the Fish, LiveFeeds + Fish and Dissolved treatment tanks, with a fish biomass of 13.8 ± 1.9 g (mean ± SD) per tank. Schools of *C. viridis* were allowed 1 week to acclimate to the experimental conditions prior to the start of the experiment, during which they were fed in the same manner as during the experiment (detailed below). A biomass of ~14 g per tank was chosen as this is similar to observed populations of damselfish that aggregate on wild coral colonies, thus providing a likely physiological response to the coral, in addition to being a reasonable holding density to ensure the welfare of the fish [15].

Ten fragments of each coral species were weighed using buoyant weight and placed into a randomised array into each of the replicate tanks, with a roughly even number of each genotype per tank. Replicate tanks were then subjected to one of the six treatments for 3 months. Each tank was supplied with 0. 1 µm filtered seawater (FSW) at 28 ± 0.1°C, at a flow-through rate of 0.8 L min^−1^, resulting in approximately one complete exchange of tank water per hour. Tanks were each fitted with a circulation pump (Turbelle nanostream 6015, Tunze Aquarientechnik, Penzberg, Germany) to enhance water circulation within the tank. Light was supplied by one LED light (Hydra, AquaIllumination, Bethlehem USA) per tank, at 150 μmol cm^−2^ s^−1^ from 0830 to 1630 with an even mix of blue and white light, with 1-h ramps at the beginning and end of the light period each day.

### 2.2. Feeds and Maintenance

Tanks containing *C. viridis* and the Pellet treatment tanks were fed twice daily at 0900 and 1530 with 0.12 g per tank of AF Tiny Fish Feed (Aquaforest, Brzesko, Poland) which had been processed briefly in a blender to reduce pellet size (≤1 mm). Live feeds for the LiveFeeds and LiveFeeds + Fish treatments consisted of PUFA-enriched *Artemia* nauplii, SELCO S.parkle-enriched rotifers and a mix of microalgae (*Nannochloropsis oceania*, *Tisochrysis lutea*, *Chaetoceros muelleri*, *Dunaliella* sp. and *Proteromonas sulcata*) fed to tanks at a rate of 0.5 nauplii mL^−1^, 0.5 rotifers mL^−1^ and 2000 cells mL^−1^. LiveFeeds and LiveFeeds + Fish feeding occurred as a daily pulse of food at 1500, after which fish were fed their afternoon pellet allotment. Tanks were cleaned and siphoned twice weekly, and trays holding coral fragments were swapped for clean trays once a week, to minimise the growth of algae around the corals. All care was taken to minimise harm to the experimental animals, following animal ethics approvals.

### 2.3. Data Collection

Growth was measured via buoyant weight (to 0.001 g), with proportional growth of coral fragments calculated by taking the raw buoyant weight of each fragment at the end of the experiment and dividing by the buoyant weight prior to the start of the experiment. Photosynthetic efficiency was measured as dark-adapted maximum quantum yield (Fv/Fm) (Walz Mini-PAM, Heinz Walz, Effeltrich, Germany).

Water samples were collected weekly from tanks, at the same time each day prior to morning fish feeding. Each experimental tank was sampled, including both the fish holding tank and coral tank for the Dissolved treatment. These samples were analysed for dissolved organic carbon (DOC) (Shimadzu TOC-L), for dissolved inorganic nutrients (NH_4_, PO_4_, NO_2_ + NO_3_, NO_2_; SEAL Analytical AutoAnalyzer) and particulate nitrogen and carbon (Shimadzu TOC-L with TNM-L and SSM). The incoming water to the tanks was analysed weekly for dissolved inorganic nutrients (SEAL Analytical AutoAnalyzer), as well as pH (HACH HQ2200 multimeter with HACH pH probe PHC101), salinity (HACH Conductivity probe CDC401) and alkalinity (Metrohm autotitrator with Dosino 800 burette). The SeaSim water supply was also tested for trace elements using IPC-OES and IPC-MS; a summary of these parameters is presented in the Supporting Information Tables S1 and S2.

At the end of the experiment, fish were anaesthetised using AQUI-S (isoeugenol, 540 mg L^−1^ AQUI-S New Zealand Ltd) and then weighed. After the final buoyant weight and photosynthetic efficiency measurements were taken, two fragments of each species from each replicate tank were randomly selected and preserved at −20˚C. The tissue from these fragments was then removed using FSW and high-pressure air and the resulting tissue blastate homogenised, ensuring the fragments and blastate were kept on ice. Tissue blasted coral skeletons were dried at 60°C and wax dipped [45] to determine surface area, which was used to standardise *Symbiodiniaceae* counts and protein content.

A Pierce BCA Protein Assay Kit was used to measure protein content within the coral blastate. A 2 mL aliquot of the homogenised tissue blastate was also taken, filtered through a 20 µm cell strainer using FSW and then fixed using 10% formalin. Subsamples of the fixed blastate were analysed in duplicate on a BD Acurri C6 Plus CSampler Flow Cytometer to determine *Symbiodiniaceae* density.

### 2.4. Data Analysis

All data analysis was conducted using RStudio version 2023.6.1.524 [46] and R version 4.3.1 [47].

Relationships between treatments and the response variables were examined using Bayesian hierarchical models using *brms* [48] and *rs*tan [49]. Model distribution and link function varied between different predictors: details are available in Supporting Information Table S3. In general, treatment was the effect, with tank replicate, genotype and/or sample as blocking factors. In all cases, weakly informative priors were used, calculated from the median and absolute median deviation of the reference group for the model. For all models, Markov chain Monte Carlo sample diagnostic outputs were checked for well-mixed traceplots, chain convergence (R-hat < 1.01), autocorrelation and effective sample size. Plots of posterior predictive checks and DHARMa residuals were then examined to determine goodness of fit for each model. Median and 95% credibility intervals (the Bayesian version of a confidence interval, calculated as 95% highest posterior density interval; see Hespanhol [50] as a guide for interpreting Bayesian credible intervals) were calculated to visualise differences in responses between treatments. The Bayesian probability that the response of one treatment was greater than the response of another treatment was is presented as *P*_*x*>*y*_ (i.e. probability that there is a true difference in the response of treatment ‘*x*' is greater than treatment ‘*y*').

## 3. Results

### 3.1. Water Quality

A summary of the modelled median and 95% credibility intervals for each water quality parameter is presented in Table 1. Ammonium (NH_4_) concentrations were 2.64 times higher (modelled median) in tanks with access to fish wastes than those without. Higher NH_4_ was recorded in the LiveFeeds + Fish treatment (0.449 µmol L^−1^) compared to Fish (0.351 µmol L^−1^), indicating some additional NH_4_ from the live feeds, though there was no difference in the concentration of NH_4_ between the Control (0.112 µmol L^−1^and LiveFeeds (0.114 µmol L^−1^) treatment tanks. Water from the Pellets treatment, however, contained slightly higher NH_4_ concentrations (0.173 µmol L^−1^) than the Control tanks. The Dissolved fish holding tanks also had a slightly higher concentration of NH_4_ (0.385 µmol L^−1^, 0.348–0.417) compared to the Dissolved coral tanks (0.229 µmol L^−1^, 0.211–0.252) that it was supplying.

The N:P ratio was the highest in LiveFeeds + Fish (10.3), followed by Fish (8.9) and the Dissolved fish tank (9.1), and was the lowest in Control (6.1). Generally, NO_2_, NO_3_ and PO_4_ were the highest in LiveFeeds + Fish tanks, followed by Fish and the Dissolved fish tanks. LiveFeeds and Pellets varied but typically had slightly higher concentrations than the Control and Dissolved coral tanks, which had the lowest concentrations of these dissolved nutrients. LiveFeeds + Fish had the lowest DOC concentration, whilst the Dissolved fish tanks had the highest, followed by the Dissolved coral tanks. DOC was relatively similar in all other treatments. Particulate C and N were likely influenced by filamentous algae observed growing in the tanks (and was visibly observed on filters during PC/N sampling) but were typically the highest in the Dissolved corals tanks, lowest in Live Feeds and relatively similar in all other tanks.

### 3.2. Growth Performance, Tissue Protein and Symbiont Metrics

#### 3.2.1. *Pocillopora verrucosa*

**Figure 1:**
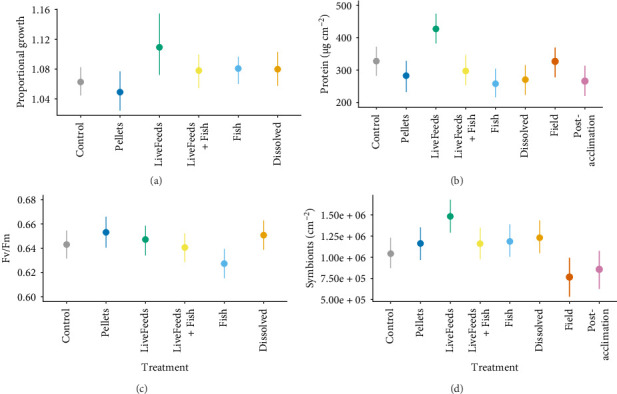
*Pocillopora verrucosa*: (a) proportional growth (buoyant weight after 3 months subjected to different dietary treatments ÷ buoyant weight at the start of the experiment), (b) tissue protein content, (c) photosynthetic efficiency measured as Fv/Fm after 3 months of exposure to treatments and (d) concentration of *Symbiodiniaceae* in tissue. Coloured points and bars represent modelled median and 95% credibility intervals.

There was no difference in the proportional growth of *P. verrucosa* between the LiveFeeds + Fish (1.078× greater buoyant weight than the initial weight 3 months after the start of experiment), Fish (1.081× greater) or Dissolved (1.080× greater) treatments (P < 85%), though all treatments exhibited higher growth compared to the Control (1.063× greater) (P_LF,F,D>C_ > 90%). *P. verrucosa* fragments exhibited the highest growth overall in the LiveFeeds treatment (1.109× greater), with evidence this was a higher growth than the Control, Pellets, Dissolved, Fish and LiveFeeds + Fish (P_L>C,P,D,F,LF_ = 92%) treatments (Figure 1a). *P. verrucosa* fragments in the Pellets treatment had much lower growth (1.049× greater) compared to all other treatments (P_D,F,L,LF>P_ > 95%) except for Control (P_C>P_ = 87%).

Tissue protein content was the highest in *P. verrucosa* fragments from the LiveFeeds treatment (427.5 µg cm^−2^); with very strong evidence, this represented an increase over the corals in the other treatments (P_L>C,D,F,LF,P_ > 99%) (Figure 1b). Control (327.9 µg cm^−2^) and LiveFeeds + Fish (297.4 µg cm^−2^) corals had the next highest protein concentrations, but the evidence suggested only Control represented an increase compared to the Dissolved (270.6 µg cm^−2^), Pellets (282.8 µg cm^−2^) and Fish (258.0 µg cm^−2^) treatments (P_C>D,P,F_ > 91%). Field fragments also had relatively high protein (327.1 µg cm^−2^), and this was higher than the Post-acclimation fragments (266.2 µg cm^−2^) (P_Fie > Pa_ = 96%).

After 3 months, there were few differences in photosynthetic efficiency between the *P. verrucosa* fragments among the treatments (Figure 1c), though Fish treatment corals tended to exhibit a lower photosynthetic efficiency compared to all other treatments (0.627 Fv/Fm) (P_C,D,L,LF,P>F_ > 90%). Symbiont densities were higher in the LiveFeeds fragments compared to all other treatments (1.48 × 10^6^ cm^−2^, P_L>C,F,D,LF,P_ > 97%) (Figure 1d). Only Dissolved *P. verrucosa* (1.23 × 10^6^ cm^−2^) were also found to have higher symbiont concentrations than the Control (P_P>C_ = 92%), whilst Fish (1.19 × 10^6^ cm^−2^), LiveFeeds + Fish (1.16 × 10^6^ cm^−2^) and Pellets (1.16 × 10^6^ cm^−2^) all had similar concentrations to the Control fragments (1.04 × 10^6^ cm^−2^). All fragments subjected to the experimental treatments had higher densities than the Field (0.77 × 10^6^ cm^−2^, 0.53–0.99) and Post-acclimation corals (0.86 × 10^6^ cm^−2^, 0.63–1.08) (P_>Fie,Pa_ > 90%).

#### 3.2.2. Acropora kenti

**Figure 2:**
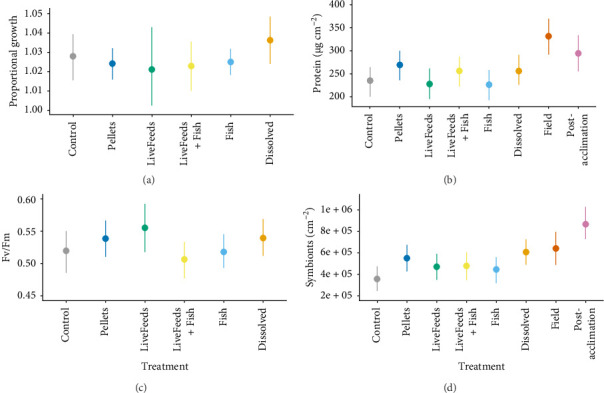
*Acropora kenti*: (a) proportional growth (buoyant weight after 3 months in treatments ÷ buoyant weight at the start of the experiment), (b) tissue protein content, (c) photosynthetic efficiency measured as Fv/Fm after 3 months ofexposure to treatments and (d) concentration of *Symbiodiniaceae* in tissue. Coloured points and bars represent modelled median and 95% credibility intervals.

*Acropora kenti* was the only species to experience significant mortality during the experiment; Dissolved treatment tanks had the highest overall survival (62.5% ± 8.5%; mean ± SE), whilst Control had the lowest (30.0% ± 5.8%), and the remaining treatments all had relatively similar survival levels (40%–53%). Growth was also the highest in Dissolved (1.036× greater), with evidence this growth was higher than all other treatments (P_D>F,L,LF,P_ > 90%) except the Control (P_D>C_ = 83%) (Figure 2a). There was no evidence for a difference in growth between Fish, LiveFeeds + Fish, LiveFeeds or Pellets or between these treatments and the Control (P < 85%).

Despite having the highest growth, fragments in the Dissolved treatment (256.4 µg cm^−2^) had similar protein concentrations to corals subjected to the Pellets (269.7 µg cm^−2^) and LiveFeeds + Fish (256.7 µg cm^−2^) treatments (P_D<P,LF_ < 85%). Control (235.6 µg cm^−2^), Fish (226.8 µg cm^−2^) and LiveFeeds (228.2 µg cm^−2^) all had slightly lower protein content compared to the other treatments. In contrast, protein was the highest in Field samples of *A. kenti* compared to Post-acclimation and all fragments from treatments (Figure 2b), and Post-acclimation fragments also had higher protein than any of the treatment corals (P < 85%).

Similar to *P. verrucosa*, there were few differences in photosynthetic efficiency between the treatments, though Fv/Fm was lower in LiveFeeds + Fish and higher in LiveFeeds (P_L>LF_ = 98%; Figure 2c). Symbiont densities were greatest in Dissolved (6.07 × 10^5^ cm^−2^) and lowest in Control fragments (3.58 × 10^5^ cm^−2^), whilst the other treatment corals had relatively similar symbiont concentrations (4.47–5.51 × 10^5^ cm^−2^) (Figure 2d). Post-acclimation (8.67 × 10^5^ cm^−2^) fragments had higher median symbiont densities than the treatment and Field corals (P > 98%), though Field corals had similar symbiont densities (6.42 × 10^5^ cm^−2^) to both Dissolved and Pellets fragments (P_F<D,P_ < 85%).

#### 3.2.3. *Porites lutea*

**Figure 3:**
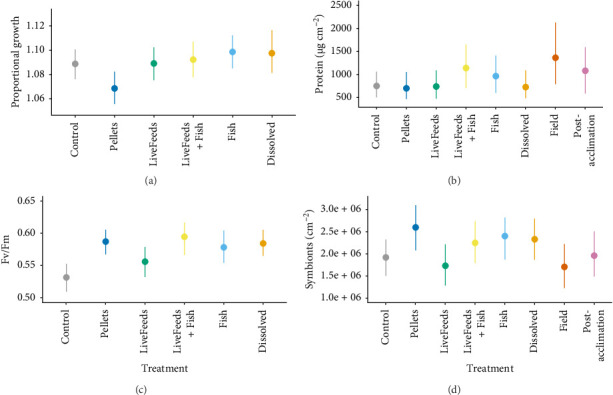
*Porites lutea*: (a) proportional growth (buoyant weight after 3 months in treatments ÷ buoyant weight at the start of the experiment), (b) tissue protein content, (c) photosynthetic efficiency measured as Fv/Fm after 3 months of exposure to treatments and (d) concentration of *Symbiodiniaceae* in tissue. Coloured points and bars represent modelled median and 95% credibility intervals.

*P. lutea* fragments exhibited the highest growth in Fish (1.099× greater), Dissolved (1.098× greater) and LiveFeeds + Fish (1.092× greater) treatments. Pellets had lower growth compared to all other treatments (1.067× greater; P_>P_ > 98%), and Control (1.089× greater) and LiveFeeds (1.089× greater) had lower growth than fragments in the Fish treatment (P_F>C,L_ > 86%; Figure 3a).

*P. lutea* fragments from the LiveFeeds + Fish (1145.3 µg cm^−2^) treatment had the highest protein concentration of the treatment corals (P_LF>_ >92%), though the Fish fragments (969.9 µg cm^−2^) and Field fragments of *P. lutea* (1368.4 µg cm^−2^) also had similar protein content P_LF>Fie,F_ < 85%). All remaining treatment fragments had median concentrations of 706–757 µg cm^−2^ (Figure 3b). Post-acclimationfragments also had higher protein content than the majority of the treatments (1086.0 µg cm^−2^), though it was not dissimilar to that observed in the LiveFeeds + Fish and Fish treatment (P_Pa<LF,F_ < 85%).

Photosynthetic efficiency was higher in the fish treatments (Dissolved, Fish and LiveFeeds + Fish) compared to the Control (P_F,LF,D>C_ > 98%). LiveFeeds also had lower photosynthetic efficiency relative to these treatments (P_F,M,D>L_ > 90%), whilst fragments in Pellets had similar levels to the three fish treatments (Figure 3c). Symbiont concentrations were elevated in the three fish treatment (2.25–2.40 × 10^6^ cm^−2^) compared to both Control (1.92 × 10^6^ cm^−2^) and LiveFeeds (1.73 × 10^6^ cm^−2^) (P_D,F,LF>C,L_ > 87%). Field (1.71 × 10^6^ cm^−2^) and Post-acclimation(1.96 × 10^6^ cm^−2^) had lower concentrations compared both Pellets and the three fish treatments (Figure 3d), though only Field corals had strong evidence of being lower (P_Fie<D,F,LF,P_ > 93%).

#### 3.2.4. *Platygyra daedalea*

**Figure 4:**
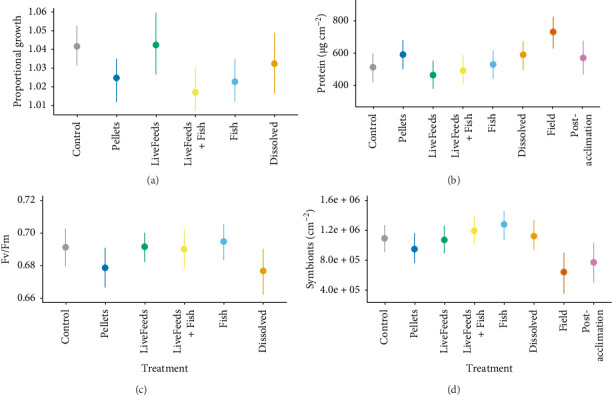
*Platygyra daedalea*: (a) proportional growth (buoyant weight after 3 months in treatments ÷ buoyant weight at the start of the experiment), (b) tissue protein content, (c) photosynthetic efficiency measured as Fv/Fm after 3 months of exposure to treatments and (d) concentration of *Symbiodiniaceae* in tissue. Coloured points and bars represent modelled median and 95% credibility intervals.

The highest growth in *P. daedalea* fragments was observed in the LiveFeeds treatment (1.042× greater). Though there was no evidence this was higher compared to the Control (1.042× greater; P_L>C_ = 53%), there was evidence this was an improvement over the LiveFeeds + Fish (1.017× greater), Fish (1.023× greater) and Pellets (1.025× greater) treatments (P_L>LF,F,P_ > 95%; Figure 4a).

Protein content was the highest in Pellets (591.3 µg cm^−2^) and Dissolved (590.2 µg cm^−2^) though this did not represent an increase compared to Control fragments (512.8 µg cm^−2^; P_D,P>C_ ≈ 84%). Fish (530.0 µg cm^−2^) and LiveFeeds + Fish (493.0 µg cm^−2^) also had similar protein levels to the Control, whilst LiveFeeds (464.3 µg cm^−2^) had the lowest concentration of all the treatment fragments (Figure 4b). Field *P. daedalea* samples had much higher protein content than any of the treatment or Post-acclimationcorals (P_Fie>_ >97%), though Post-acclimationfragments had similar levels to both Dissolved and Pellets (570.6 µg cm^−2^).

Conversely, whilst photosynthetic efficiency was similar in Pellets and Dissolved, it was lower than the other treatments (P_C,F,LF,L>P,D_ > 90%). The other treatments had similar efficiency levels, though corals in the Fish treatment displayed the highest overall photosynthetic efficiency (Figure 4c). Symbiont concentrations were also the highest in the Fish (1.28 × 10^6^ cm^−2^) and LiveFeeds + Fish (1.19 × 10^6^ cm^−2^) fragments (Figure 4d), though only Fish corals had consistence evidence of concentrations being higher than other treatment corals (P_F>C,D,L,P_ > 87%). Dissolved (1.12 × 10^6^ cm^−2^), Control (1.09 × 10^6^ cm^−2^) and LiveFeeds (1.07 × 10^6^ cm^−2^) all had slightly lower concentrations compared to these treatments, with the Pellets (0.95 × 10^6^ cm^−2^) having the lowest of all the treatment corals. Field and Post-acclimation also had lower symbiont concentrations compared to all the *P. daedalea* fragments in the treatments (P_>Fie,Pa_ > 85%).

## 4. Discussion

### 4.1. Coral Species Varied Responses to Treatments

In this study, corals were exposed to various isolated or combined dissolved and particulate wastes from schooling damselfish. Whilst the response of the different coral species varied, all species displayed some positive responses to the presence of fish wastes. For example, tissue protein and symbiont densities were higher in *A. kenti* and *P. lutea* with access to dissolved fish wastes, with growth also slightly enhanced in *P. lutea* under these treatments. Whilst live feeds resulted in the highest growth and protein content for the more heterotrophic *P. verrucosa*, the presence of fish or dissolved fish wastes could increase growth and symbiont concentrations in this species compared to unfed controls. *P. daedalea* had a more mixed response but did also have higher symbiont and protein densities in treatments with dissolved fish wastes. Coral growth and other physiological responses will be dependent upon culturing conditions, including culture systems, water flow, water quality, handling and feeds, which will vary between facilities. In this study, we focussed on comparisons between the treatments and the controls, looking for changes based on the presence/absence of fish and/or feeds, which provide useful baseline comparisons for facilities to further explore in their individual culture systems.

Coral fragments performed well in both LiveFeeds + Fish and Fish treatments (where fish were kept with the corals) and the Dissolved treatment (where corals only had access to dissolved fish wastes), suggesting that benefits were primarily from the dissolved portion of fish waste. Though there was little evidence corals directly fed on fish faeces, it has been suggested that leeching from faecal pellets could still be an important source of nitrogen or phosphorous for corals [13, 51]. Higher protein and symbiont concentrations in corals due to association with fish are in line with previous studies on fish–coral interactions [20, 22], as is the increased growth observed in *P. lutea* and *P. verrucosa* in the present study [14, 17, 18].

### 4.2. Effects of Nutrient Enrichment by Fish

Treatments with access to fish waste were associated with increased nitrogen and phosphorous availability, particularly NH_4_ which is the coral's preferred form of inorganic nitrogen [52]. Comparing average NH_4_ concentrations in the incoming FSW from the SeaSim system (Supporting Information Table S1) to the Control tanks, we observed a decrease of ~0.07 µmol L^−1^, the majority of which is likely due to uptake by the coral's symbionts [53]. Similarly comparing the Dissolved treatment ‘fish only' tanks to the ‘coral only' tanks supplied with the fish's wastewater, we observe a ~0.16 µmol L^−1^ decrease in NH_4_ concentration in the ‘coral only' tanks, potentially indicating enhanced uptake of NH_4_ from the fish wastes. NO_3_ concentrations were inversely affected, with Control tanks showing an ~0.52 µmol L^−1^ decrease compared to the incoming SeaSim water, whilst ‘coral only' tanks showed an ~0.38 µmol L^−1^ decrease compared to the ‘fish only' tanks. Though we did not directly measure the uptake of nutrients by the corals, it does indicate that the corals and their symbionts were modulating their nitrogen uptake in response to the fish wastes. Potential PO_4_ was also slightly higher in the ‘coral only' tanks with fish wastewater (~0.04 µmol L^−1^) compared to the Control tanks (0.02 µmol L^−1^), though these differences were minor.

The changes in nutrient profiles and sources likely influenced the responses observed in the present study, particularly relating to changes in photobiont concomitant with moderate nitrogen levels [52, 54–58]. Carbon-rich photosynthates from algal symbionts can often meet the energy demands and sustain growth of corals, and excess symbiont cells can be digested by coral hosts in order to uptake important nutrients like nitrogen and phosphorous [38, 59]. High nitrogen availability with phosphorous limitation can disturb the coral–algae symbiosis, leading to increased susceptibility to bleaching [60, 61], though high NH_4_ enrichment with simultaneous high PO_4_ enrichment can increase the density of symbionts whilst also decreasing the translocation of fixed carbon from symbionts to the coral host [52, 62]. Enrichment of both nitrogen and phosphorous at moderate levels has been shown to increase carbohydrate and nitrogen content of coral tissue [38, 63], with a N:P ratio of 4–7 considered normal for Great Barrier Reef waters [60].

In this experiment, treatments with fish displayed a higher N:P ratio, that is, 10.3 in LiveFeeds + Fish versus 6.9 in the Control. There was also evidence for PO_4_ enrichment from both LiveFeeds and the treatments with fish. As such, most corals in the experiment likely experienced moderate N and P enrichment, with *P. verrucosa* and *P. lutea* demonstrating higher growth and symbiont densities in treatments with high N:P ratios (Dissolved, Fish and LiveFeeds + Fish) than Control corals. Photosynthetic rates were also observed to be within normal ranges for all corals [64], suggesting that for *P. verrucosa* and *P. lutea*, the supply of fixed carbon from symbionts was not adversely affected by N and P enrichment, and the higher symbiont densities may represent an increased food supply for the corals as they may ‘farm' and digest the excess algal cells [38].

However, high symbiont density does not necessarily correspond to ubiquitous high growth. *P. daedalea* subjected to treatments with fish displayed the highest symbiont concentrations yet recorded the lowest growth. Similarly, *A. kenti* demonstrated higher symbiont densities in the Dissolved treatment, but the rates of growth remained similar to the low-symbiont density Control corals. The observed N:P levels were potentially optimal for *P. lutea* and *P. verrucosa*, though for *P. daedalea* and *A. kenti* they potentially compromised carbon exchange with the symbionts, demonstrating the importance of considering species specific responses of corals to nutrient availability [52, 63]. Similarly, *A. kenti* was the only coral with high rates of mortality during the experiment, though given there was relatively high mortality (>30%) across all treatments, potentially the holding conditions were not ideal for this species.

Interestingly, there were few differences in photosynthetic efficiency (Fv/Fm) between the treatments for any of the coral species; in contrast to previous studies, they found that under ambient conditions, fish could induce higher photosynthetic rates in their coral hosts [19–21]. However, these previous published experiments were done under lower flow conditions, and in the case of Garcia-Herrera et al. [19] with small colonies, whilst the present experiment used fragments and circulation pumps to remove the possible effects of oxygenation from fish fanning [23, 24]. This could account for the limited response of photosynthetic efficiency to the fish, as previous research has shown increased flow can drive increases in coral photosynthesis; thus, our higher flow may have dampened any influence from the fish [65].

All coral species in the present experiment recorded different protein and symbiont densities compared to those from the field and those acclimated to the facility prior to the commencement of the experiment; however, it should be noted that wild corals do experience natural, seasonal fluctuations in various physiological parameters including their nutritional composition [66, 67]. General increases in symbiont density between the field to the captive-acclimated corals were likely driven by acclimation to the lower light conditions compared to the field [68, 69], increased nitrogen [55, 56, 70] and food availability [71]. Protein was also higher in field corals compared to captive corals, apart from *P. verrucosa* which had a higher protein concentration in the LiveFeeds treatment. Protein is typically higher at sites of growth in corals [72], though higher growth rates are not always associated with elevated protein concentrations [33], and concentrations may be reduced if the dietary protein supply is inadequate, where protein may be transported from less vital areas of the coral and subsequently catabolised to maintain physiological function [73]. Nevertheless, it is likely that the higher protein, for example, in *P. verrucosa* fragments in the LiveFeeds treatment, was due to the higher growth of in these corals, whilst the lower concentration of the other corals compared to the Field samples may be a result of protein depletion due to a suboptimal supply of dietary protein.

### 4.3. The Influence of Heterotrophic Feeding

Whilst the presence of fish did enhance the growth of *P. verrucosa* fragments, the highest growth for this species was observed in the LiveFeeds treatment. This genus has been identified as comparatively more heterotrophic than *Acropora* or *Porites* [28], which would explain the greater growth, protein and symbiont concentration observed when given access to live feeds. Past research has shown that even in high light environments, more heterotrophic corals with access to heterotrophic food sources will grow faster and have higher symbiont and protein concentrations compared to unfed colonies [33, 36, 71, 74]. Interestingly, there was no evidence for differences between the *P. verrucosa* in the LiveFeeds + Fish treatment (which also had access to live feeds) and the Fish or Dissolved treatment. A likely explanation for this is the *C. viridis* in the tank were opportunistically feeding on the supplied live feeds before the corals were able to capture them, resulting in a lower food availability for the corals. *P. daedalea* have also been identified as more heterotrophic than other coral species [29], but there was no evidence for a difference in growth rates between the Control and LiveFeeds treatments. *Platygyra* corals have been noted to be tolerant to a wide range of conditions [29, 64, 75] and exhibit relatively slow growth [76]; thereby, it may be that the experimental conditions where not sufficient to create a substantial change in growth rates for this species.

Across all coral species in the present experiment, the feeding of fish pellets to coral fragments directly resulted in reduced growth compared to the other feed treatments and the unfed control. Some tissue loss was also observed in fragments from this treatment, consistent with the Osinga et al. [77] observation that batch feeding with dry food could potentially harm corals. The use of batch feeds to provide optimal nutrition for captive corals remains a vexing topic for researchers and aquarists, though it is obvious that at least some level of heterotrophic feeding or nutrient enrichment is necessary to enhance production performance [36].

### 4.4. Considerations for Integrating Fish Into Large-Scale Coral Aquaculture

Long-term impacts of nutrient enrichment on corals should be considered when introducing fish into coral aquaculture systems. This experiment demonstrated some benefits from moderate N:P enrichment, though chronic nutrient enrichment can have both positive and negative effects, depending on the level of enrichment [78–80]. Such responses are complex and often species specific, even within closely related coral species; thus, commercial coral propagation must carefully consider what nutrient profiles are appropriate to support the range of species they culture. Similarly, the level of the nutrient enrichment from the presence of fish will depend on how the water in the system is retained or treated. Most recirculating coral aquaculture systems are equipped with biological and mechanical filtration as well as foam fractionation to control excess nutrients. Here, we utilised a relatively high turnover flow-through system; thus, NH_4_ concentrations may be lower in recirculating systems that use biological filtration to facilitate nitrification. Therefore, coral aquaculture facilities should implement robust, long-term water quality monitoring to identify and modulate nutrient levels influenced by companion fish.

NH_4_ is the symbionts' preferred form of inorganic nitrogen, whilst NO_3_ from anthropogenic sources is less favoured [52]. Whilst NH_4_ and PO_4_ can also be dosed using sources such as readily available inorganic liquid fertilisers, previous studies have shown corals generally respond better to natural sources of these nutrients [58]. Thus, incorporating fish into coral culture may be a convenient method of provisioning coral with a more balanced nutrient profile (N/P) to maintain holobiont health. Additionally, fish nutrients and artificial dosing could be used in conjunction to achieve a desired nutrient profile, like the paired application of chemical treatments and cleaner fish to control parasitic copepods in salmon aquaculture [81, 82].

Though the direct effects of fish coculture on coral growth performance and symbiont concentration may vary depending on species and husbandry conditions, the application of fish in coral aquaculture can have indirect benefits. Fish such as *Pseudocheilinus hexataenia* have been shown to be potential biological controls for coral parasites like *Prosthiostomum* flatworms [83]. Similarly, herbivorous species such as acanthurids are also used as algae controllers both in ex situ facilities where they are deliberately added to aquaria [84, 85] or in situ nurseries where they may be released or naturally recruit [86–88]. As such, it is worth considering the potential roles fish could play in coculture scenarios, both as pest and/or algae controllers and as a source of nutrient enrichment. Based on our results, 14 g of fish biomass per 50 L with twice daily feeds for the fish could be suggested as an initial baseline density of fish in coral culture systems, with subsequent adjustments depending on which species of fish is used and the requirements of different facilities' culture systems.

## 5. Conclusions

Overall, our study has demonstrated that integrated fish–coral culture has the potential to enhance physiological traits of cultured corals by supplying a source of moderate nutrient enrichment. However, the response of corals to fish co-culture varies markedly between genera; thus, producers must carefully consider the nutrient requirements of their targeted coral species before integrating fish into culture. The baseline of 14 g of fish biomass per 50 L of tank water provides a preliminary target for producers, though it needs to be tailored to different corals' requirements and to the variable nutrient output of different fish species if introducing fish companions to serve other functions, for example, algae removal. Ultimately, integrating fish into coral culture represents a potential efficient, minimal husbandry method to provide enrichment to cultured corals, which will improve productivity to meet the growing demands of both reef restoration and the ornamental industry. Future research should assess a wider variety of coral and fish associations, exploring the underlying physiological effects of fish coculture on corals to better understand the drivers of their species-specific responses.

## Figures and Tables

**Table 1:** Water quality from treatment tanks during experiment (median and 95% CI [HPDI] from Bayesian hierarchical models).

Treatment	NH_4_ (μmol L^−1^)	NO_2_ (μmol L^−1^)	NO_3_ (μmol L^−1^)	PO_4_ (μmol L^−1^)	DOC (mg L^−1^)	PC (μg L^−1^)	PN (μg L^−1^)	N:P
Control	0.112 0.102–0.122	0.049 0.044–0.052	0.917 0.824–1.028	0.169 0.161–0.176	1.13 1.10–1.16	54.29 49.64–58.69	11.55 10.53–12.66	6.1 5.8–6.5
Pellets	0.173 0.157–0.189	0.057 0.052–0.063	1.153 1.023–1.284	0.171 0.164–0.179	1.11 1.09–1.14	57.31 52.64–62.55	12.76 11.63–14.07	7.8 7.3–8.3
LiveFeeds	0.114 0.103–0.124	0.053 0.048–0.058	1.047 0.927–1.165	0.178 0.170–0.186	1.13 1.10–1.16	45.47 42.09–48.88	9.67 8.77–10.61	6.6 6.2–7.0
LiveFeeds + Fish	0.449 0.410–0.491	0.058 0.052–0.063	1.419 1.265–1.580	0.184 0.176–0.192	1.07 1.04–1.10	52.95 49.52–57.37	10.57 9.53–11.56	10.3 9.7–10.9
Fish	0.351 0.322–0.383	0.056 0.051–0.061	1.223 1.084–1.358	0.179 0.170–0.187	1.13 1.10–1.17	55.68 50.58–60.68	11.60 10.48–12.68	8.9 8.4–9.5
Dissolved Coral tank	0.229 0.211–0.252	0.050 0.045–0.054	0.849 0.764–0.955	0.141 0.133–0.149	1.16 1.13–1.20	64.01 57.69–70.28	13.75 12.50–15.15	7.5 7.1–8.0
Dissolved Fish tank	0.385 0.348–0.417	0.051 0.046–0.056	1.229 1.107–1.379	0.175 0.166– 0.182	1.22 1.18–1.26	58.59 52.83–64.20	11.74 10.61–12.83	9.1 8.6–9.7

## Data Availability

Data and code are available via https://github.com/blue-bio/coral_fish_nutrition_public. Data are also available via the AIMS Data Centre https://doi.org/10.25845/2SWS-G533.

## Supporting Information

Supporting Information The supporting information of this manuscript provides a more detailed description ofthe adapted BCA protein assay methodology used for the coral tissue analysis, results for the water quality tests of the incoming filtered seawater and details of the parameters for each Bayesian hierarchical model. Table S1: Water quality of incoming filtered seawater to experimental tanks. Table S2: Trace elements testing of Sea Simulator filtered seawater for experimental systems. Table S3: Bayesian hierarchical model parameters.
