# Supplementary material for: Improving Coral Grow-Out Through an Integrated Aquaculture Approach
Source: Aquac Nutr. 2025 Apr 13;2025:1446195. doi: 10.1155/anu/1446195 (PMC12009681; doi:10.1155/anu/1446195)
Supplement: Supporting Information — The supporting information of this manuscript provides a more detailed description ofthe adapted BCA protein assay methodology used for the coral tissue analysis, results for the water quality tests of the incoming filtered seawater and details of the parameters for each Bayesian hierarchical model. Table S1: Water quality of incoming filtered seawater to experimental tanks. Table S2: Trace elements testing of Sea Simulator filtered seawater for experimental systems. Table S3: Bayesian hierarchical model parameters. [file 1446195.f1.docx]

**Improving coral grow-out through an integrated aquaculture approach: supplementary materials**

Rachel C. Neil^1,2,3*^, Jonathan A. Barton^2^, Andrew Heyward^4^, David S. Francis^5^, Leo Nankervis^1^, Thomas S. Mock^5^, David G. Bourne^1,2^ & Craig Humphrey^2^

^1^College of Science and Engineering, James Cook University, 1 Angus Smith Drive, Douglas, QLD 4814, Australia

^2^Australian Institute of Marine Science, Cape Cleveland, QLD 4811, Australia



^3^AIMS@JCU, James Cook University, DB17-148, Townsville, QLD 4811, Australia

^4^Australian Institute of Marine Science, Indian Ocean Marine Research Centre, University of Western Australia, 39 Fairway Street, Crawley, WA 6009, Australia

^5^Nutrition and Seafood Laboratory (NuSea.Lab), School of Life and Environmental Sciences, Deakin University, Geelong, Queenscliff Marine Station, 2A Bellarine Highway, Queenscliff, Victoria, 3225

*Corresponding author: rachel.neil@my.jcu.edu.au

**BCA Protein Assay method adapted for coral samples**

A Pierce™ BCA Protein Assay Kit was used to determine protein content within the coral tissue. 500µL of homogenised tissue blastate was taken and mixed with 500µL 0.5M NaOH, then incubated at 60˚C for 5 hours. In duplicate, 25 µL of incubated sample and 200 µL working reagent from the kit was then added to a well of 96-well plate, agitated horizontally, then incubated in darkness at 37˚C for 30 mins. The plates were allowed to cool for 5 minutes, then absorbance at 562nm measured. Using standards of known protein concentration a standard curve was constructed, and the protein concentration within each of the samples calculated along it.

| Supplementary Table 1: Water quality of incoming filtered seawater to experimental tanks  (Mean ± standard deviation) | | | | | | |
| --- | --- | --- | --- | --- | --- | --- |
| Salinity  (ppt) | NH_4_  (µmol L^-1^) | NO_2_  (µmol L^-1^) | NO_3_  (µmol L^-1^) | PO_4_  (µmol L^-1^) | pH | Alkalinity  (µmol kg^-1^) |
| 34.9 ± 0.3 | 0.18 ± 0.07 | 0.12 ± 0.05 | 1.44 ± 0.51 | 0.19 ± 0.03 | 8.17 ± 0.06 | 2323 ± 25 |

| Supplementary Table 2: Trace elements testing of Sea Simulator filtered seawater for experimental systems  (Mean ± standard deviation)* | | | | | | | | | | | | |
| --- | --- | --- | --- | --- | --- | --- | --- | --- | --- | --- | --- | --- |
| Aluminium (µg L^-1^) | Barium  (µg L^-1^) | Boron  (µg L^-1^) | Bromine (mg L^-1^) | Cadmium (µg L^-1^) | Calcium (mg L^-1^) | Chromium (µg L^-1^) | Cobalt  (µg L^-1^) | Copper (µg L^-1^) | Iodine (mg L^-1^) | Iron  (µg L^-1^) | Lead  (µg L^-1^) | Lithium (µg L^-1^) |
| <5 | 9 ± 3 | 4002 ± 338 | 70 ±7 | <0.2 | 434 ± 18 | <0.5 | <0.2 | <1 | <1.0 | <5 | <0.2 | 168 ± 23 |
| Magnesium (mg L^-1^) | Manganese (µg L^-1^) | Molybdenum (µg L^-1^) | Nickel  (µg L^-1^) | Potassium (mg L^-1^) | Silicon (mg L^-1^) | Selenium (µg L^-1^) | Strontium (µg L^-1^) | Tin  (µg L^-1^) | Titanium (µg L^-1^) | Vanadium (µg L^-1^) | Zinc  (µg L^-1^) |  |
| 1382 ± 110 | <0.5 | 9 ± 2 | <1 | 425 ± 30 | <0.50 | <2 | 7542 ± 493 | <5 | <5 | 0.8 ± 0.7 | <5 |  |

* Less than values (e.g. <5) were readings below the indicated limit of detection.

| Supplementary Table 3: Bayesian hierarchical model parameters | | | | | | | |
| --- | --- | --- | --- | --- | --- | --- | --- |
| Response | Coral | Model form | Distribution | Link | Priors | Iterations (warmup) | Thinning |
| Proportional Growth | *Pocillopora verrucosa* | prop_monthly ~ treatment*month + (treatment\|replicate)  shape ~ treatment*month*genotype | Gamma | log | prior(normal(0.024, 0.1), class = 'Intercept') +  prior(normal(0, 0.1), class = 'b') +  prior(student_t(3, 0, 0.1), class = 'sd') +  prior(student_t(3, 0, 0.1), class = 'b', dpar = 'shape') | 10,000 (2,500) | 10 |
|  | *Acropora kenti* | prop_monthly ~ treatment*month + (treatment\|replicate:genotype)  shape ~ treatment*month | Gamma | log | prior(normal(0.020, 0.1), class = 'Intercept') +  prior(normal(0, 0.1), class = 'b') +  prior(student_t(3, 0, 0.1), class = 'sd') +  prior(student_t(3, 0, 0.1), class = 'b', dpar = 'shape') | 10,000 (2,500) | 10 |
|  | *Porites lutea* | prop_monthly ~ treatment*month + (treatment\|replicate:genotype)  shape ~ treatment*month | Gamma | log | prior(normal(0.045, 0.1), class = 'Intercept') +  prior(normal(0, 0.1), class = 'b') +  prior(student_t(3, 0, 0.1), class = 'sd') +  prior(student_t(3, 0, 0.1), class = 'b', dpar = 'shape') | 10,000 (2,500) | 10 |
|  | *Platygyra daedalea* | prop_monthly ~ treatment*month + (treatment\|replicate)  shape ~ treatment*month | Gamma | log | prior(normal(0.012, 0.1), class = 'Intercept') +  prior(normal(0, 0.1), class = 'b') +  prior(student_t(3, 0, 0.1), class = 'sd') +  prior(student_t(3, 0, 0.1), class = 'b', dpar = 'shape') | 10,000 (2,500) | 10 |
| Photosynthetic efficiency  (Fv/Fm) | *Pocillopora verrucosa* | Y_na ~ treatment*month + (1\|tank_rep_rand:genotype)  sigma ~ month | Gaussian | identity | prior(normal(0.7, 0.03), class = 'Intercept') +  prior(normal(0, 0.2), class = 'b') +  prior(student_t(3, 0, 0.03), class = 'sigma') +  prior(student_t(3, 0, 0.03), class = 'sd') | 5,000 (2,500) | 5 |
|  | *Acropora kenti* | Y_na ~ treatment*month + (1\|tank_rep_rand:genotype)  phi ~ treatment*month | Beta | logit | prior(normal(0.77, 0.2), class = 'Intercept') +  prior(normal(0, 0.8), class = 'b') +  prior(student_t(3, 0, 0.2), class = 'sd') +  prior(student_t(3, 0, 0.2), class = 'b', dpar = 'phi') | 7,000 (2,500) | 5 |
|  | *Porites lutea* | Y_na ~ treatment*month + (1\|tank_rep_rand)  phi ~ month*treatment*genotype | Beta | logit | prior(normal(0.44, 0.2), class = 'Intercept') +  prior(normal(0, 0.8), class = 'b') +  prior(student_t(3, 0, 0.2), class = 'sd') +  prior(student_t(3, 0, 0.2), class = 'b', dpar = 'phi') | 7,000 (2,500) | 10 |
|  | *Platygyra daedalea* | Y_na ~ treatment*month + (1\|tank_rep_rand)  sigma ~ treatment*month*genotype | Gaussian | identity | prior(normal(0.70, 0.03), class = 'Intercept') +  prior(normal(0, 0.3), class = 'b') +  prior(student_t(3, 0, 0.03), class = 'sd') +  prior(student_t(3, 0, 0.03), class = 'b', dpar = 'sigma') | 10,000 (4,000) | 10 |
| Protein  (µg c­m^-2^) | *Pocillopora verrucosa* | protein_ugSA ~ treatment + (1\|tank_rep_rand) | Gaussian | identity | prior(normal(315, 12), class = 'Intercept') +  prior(normal(0, 300), class = 'b') +  prior(student_t(3, 0, 12), class = 'sd') +  prior(student_t(3, 0, 12), class = 'sigma') | 5,000 (2,500) | 5 |
|  | *Acropora kenti* | protein_ugSA ~ treatment + (1\|tank_rep_rand) | Gaussian | identity | prior(normal(292, 42), class = 'Intercept') +  prior(normal(0, 180), class = 'b') +  prior(student_t(3, 0, 42), 'sigma') +  prior(student_t(3, 0, 42), class = 'sd') | 7,000 (2,500) | 5 |
|  | *Porites lutea* | protein_ugSA ~ treatment + (1\|tank_rep_rand) | Gamma | log | prior(normal(7, 0.8), class = 'Intercept') +  prior(normal(0, 1), class = 'b') +  prior(student_t(3, 0, 0.6), class = 'sd') + prior(gamma(0.01, 0.01), class = 'shape') | 8,000 (2,500) | 10 |
|  | *Platygyra daedalea* | protein_ugSA ~ treatment + (1 \| tank_rep_rand) | Gamma | log | prior(normal(484, 160), class = 'Intercept') +  prior(normal(0, 700), class = 'b') + prior(student_t(3, 0, 160), 'sigma') + prior(student_t(3, 0, 160), class = 'sd') | 10,000 (2,500) | 10 |
| Symbionts  (c­m^-2^) | *Pocillopora verrucosa* | zoox_SA ~ treatment + (1 \| tank_rep_rand) | Gaussian | identity | prior(normal(1100000, 125000), class = 'Intercept') +  prior(normal(0, 1000000), class = 'b') +  prior(student_t(3, 0, 125000), 'sigma') +  prior(student_t(3, 0, 125000), class = 'sd' | 6,000 (3,000) | 5 |
|  | *Acropora kenti* | zoox_SA ~ treatment + (1\|tank_rep_rand) | Gaussian | identity | prior(normal(323000, 150000), class = 'Intercept') +  prior(normal(0, 700000), class = 'b') +  prior(student_t(3, 0, 150000), 'sigma') +  prior(student_t(3, 0, 150000), class = 'sd') | 5,000 (2,500) | 5 |
|  | *Porites lutea* | zoox_SA ~ treatment + (1\|tank_rep_rand) | Gaussian | identity | prior(normal(1650000, 1400000), class = 'Intercept') +  prior(normal(0, 1100000), class = 'b') +  prior(student_t(3, 0, 1100000), 'sigma') +  prior(student_t(3, 0, 1100000), class = 'sd') | 5,000 (2,500) | 5 |
|  | *Platygyra daedalea* | zoox_SA ~ treatment + (1\|tank_rep_rand) | Gaussian | identity | prior(normal(1100000, 260000), class = 'Intercept') +  prior(normal(0, 700000), class = 'b') +  prior(student_t(3, 0, 260000), 'sigma')+  prior(student_t(3, 0, 260000), class = 'sd') | 5,000 (2,500) | 5 |
| NH_4_  (μmol L^-1^) | na | NH4 ~ treatment + (1\|tank_rep_rand:sample) | Gamma | log | prior(normal(-2.1, 0.5), class = 'Intercept') +  prior(normal(0, 1.5), class = 'b') +  prior(student_t(3, 0, 0.5), class = 'sd') +  prior(gamma(0.01, 0.01), class = 'shape' | 10,000 (2,500) | 10 |
| NO_2_  (μmol L^-1^) | na | NO2 ~ treatment + (1 \| tank_rep_rand:sample) | Gamma | log | prior(normal(-3.22, 0.9), class = 'Intercept') +  prior(normal(0, 0.8), class = 'b') +  prior(student_t(3, 0, 0.5), class = 'sd') +  prior(gamma(2, 1), class = 'shape') | 5,000 (2,500) | 5 |
| NO_3_  (μmol L^-1^) | na | NO3 ~ treatment + (1\|tank_rep_rand:sample) | Gamma | log | prior(normal(-0.332, 0.6), class = 'Intercept') +  prior(student_t(3, 0, 0.9), class = 'b') +  prior(student_t(3, 0, 0.4), class = 'sd') +  prior(gamma(2, 1), class = 'shape') | 10,000 (2,500) | 5 |
| PO_4_  (μmol L^-1^) | na | PO4 ~ treatment + (1\|tank_rep_rand:sample) | Gaussian | identity | prior(normal(0.17, 0.05), class = 'Intercept') +  prior(normal(0, 0.07), class = 'b') +  prior(student_t(3, 0, 0.05), class = 'sd') +  prior(student_t(3, 0, 0.05), class = 'sigma') | 10,000 (2,500) | 10 |
| DOC  (mg L^-1^) | na | DOC ~ treatment + (1\|tank_rep_rand:sample)  sigma ~ treatment | Shifted Log-normal | identity | prior(normal(-1.8, 0.6), class = 'Intercept') +  prior(normal(0, 0.5), class = 'b') +  prior(student_t(3, 0, 0.1), class = 'sd') +  prior(student_t(3, 0, 0.1), class = 'b', dpar = 'sigma') +  prior(uniform(0, 0.91), class = 'ndt') | 10,000 (2,500) | 10 |
| PC  (μg L^-1^) | na | PC ~ treatment + (1\|tank_rep_rand:sample)  shape ~ treatment | Gamma | log | prior(normal(2.56, 0.8), class = 'Intercept') +  prior(normal(0, 0.5), class = 'b') +  prior(student_t(3, 0, 0.8), class = 'sd') +  prior(normal(0, 1), class = 'b', dpar = 'shape') | 10,000 (2,500) | 10 |
| PN  (μg L^-1^) | na | PN ~ treatment + (1\|tank_rep_rand:sample) | Gamma | log | prior(normal(1.06, 0.6), class = 'Intercept') +  prior(normal(0, 0.6), class = 'b') +  prior(student_t(3, 0, 0.5), class = 'sd') + prior(gamma(0.01, 0.01), class = 'shape') | 10,000 (2,500) | 10 |
| N:P | na | NP ~ treatment + (1\|tank_rep_rand:sample) | Gamma | log | prior(normal(1.87, 0.5), class = 'Intercept') +  prior(normal(0, 1), class = 'b') +  prior(student_t(3, 0, 0.5), class = 'sd') +  prior(gamma(0.01, 0.01), class = 'shape') | 10,000 (2,500) | 10 |
